# Crystalloid and colloid preload for maintaining cardiac output in elderly patients undergoing total hip replacement under spinal anesthesia

**DOI:** 10.1016/S1674-8301(11)60024-9

**Published:** 2011-05

**Authors:** Rufeng Xie, Lizhong Wang, Hongguang Bao

**Affiliations:** a Department of Anesthesiology, Nanjing First Hospital Nanjing, Jiangsu 210006, China; b Department of Anesthesiology, Jiaxing Maternity and Child Care Hospital, Jiaxing, Zhejiang 314000, China

**Keywords:** anesthesia, spinal, cardiac output, aged, arthroplasty, replacement, hip

## Abstract

The aim of the present study was to compare the effects of colloid and crystalloid preload on cardiac output (CO) and incidence of hypotension in elderly patients under spinal anesthesia (SA). A randomized, double-blinded study was conducted including 47 elderly patients undergoing scheduled total hip replacement (THR), who were randomized to three groups: the control group (C group, *n* = 15), crystalloid (RS group, *n* =16) and colloid group (HES group, *n* = 16). An intravenous preload of 8 mL/kg of either lactated Ringer's solution in the RS group or 6% hydroxyethyl starch in the HES group was infused within 20 min before SA induction, while no intravenous preload was given in the C group. There was a trend of decrease in CO and systolic blood pressure after SA with time in the C group. In the RS and HES groups, CO increased significantly after fluid preloading as compared with baseline (*P* < 0.01). Thereafter, CO remained higher than baseline until 30 min after SA in the HES group. The change of systolic blood pressure was similar to CO, but no significant difference from baseline was observed in each group. Hypotension occurred in 3 patients in the C group and one each in the RS and HES group, respectively (*P* = 0.362). Intravascular volume preload with colloid is more effective than crystalloid solution in maintaining CO, which may be improved the hemodynamic stability in elderly patients during SA.

## INTRODUCTION

Hip fracture is a common disease in the elderly and often requires surgical treatment such as total hip replacement[1],[2]. However, there are predictable physiological and metabolic changes in elderly patients that limit their ability to respond to surgical and anesthetic stress. Furthermore, comorbidities such as cardiovascular disease increase with age and may contribute to perioperative adverse events and mortality[3]–[5].

The selection of anesthesia for total hip replacement can either be general or regional anesthesia. Studies have shown that regional anesthesia for total hip replacement results in better postoperative outcomes, including improved respiratory function, less nausea and vomiting, less pain, and lower incidence of deep vein thrombosis[6]–[8]. Among regional anesthetic techniques, spinal anesthesia has become the preferred technique because it offers a fast, profound, and high quality sensory and motor block with reduced risk of local anesthetic toxicity. However, hypotension remains the most common complication after spinal anesthesia and may increase the risk of myocardial ischaemia, especially in the elderly patients[3],[9],[10]. To prevent spinal anesthesia-induced hypotension, intravenous fluid preload is commonly used. However, based on previous studies in which only blood pressure and heart rate were used to assess the efficacy of fluid preload, the type and amount of fluid preload for preventing spinal hypotension are still controversial[11]-[13].

On the other hand, the true goal of hemodynamic management during anesthesia is to maintain tissue perfusion. Cardiac output has been documented to be a better indicator of cardiac function and tissue perfusion than arterial blood pressure[14]. Thus, a better understanding of cardiac output changes during spinal anesthesia would help improve hemodynamic stability in elderly patients. The aim of this randomized controlled study was to compare the effects of crystalloid and colloid preload on cardiac output (CO) during spinal anesthesia (SA) in elderly patients undergoing scheduled total hip replacement using the FloTrac/Vigileo system.

## MATERIALS AND METHODS

### Patients

The study protocol was approved by the medical ethics committee of Nanjing First Hospital and a written informed consent was obtained from all study participants. We recruited a total of 48 American Society of Anesthesiologists (ASA) physical status I-III patients (age > 60 years) scheduled for total hip replacement under SA between September 2009 and June 2010 at Nanjing First Hospital. Exclusion criteria included congestive heart failure (NYHA III-IV), unstable angina, significant aortic stenosis, recent myocardial infarction or severe cardiac arrhythmia, and contraindications to spinal anesthesia. Patients were randomly allocated to one of three groups, the control (C group), the crystalloid (RS group) and the colloid group (HES group) by computer-generated codes that were kept in sealed, numbered envelopes.

### Anesthesia

Patients were premedicated with intramuscular administration of luminal sodium 0.1 g and atropine 0.5 mg. A 20-gauge intravenous catheter was inserted into the forearm vein, and an intravenous preload of 8 mL/kg of either lactated Ringer's solution in the RS group or hydroxyethyl starch solution (6% HES 130/0.4; Voluven, Fresenius Kabi, Homburg, Germany) in the HES group was infused over 20 min before SA while no fluid preload was given in the C group. Thereafter, all patients received intravenous lactated Ringer's solution at approximately 2 mL/(kg·h) except for the additional volume for blood loss during the operation.

Once preloading was completed, spinal anesthesia was performed at the L3–L4 or L4–L5 intervertebral space using a 25-gauge pencil-point needle with the patient placed in the lateral position. After clear, free flow of the cerebrospinal fluid was obtained, 2 mL hyperbaric 0.5% bupivacaine (10 mg) was injected intrathecally over 10 s. The patient was then immediately turned to the supine position. The upper sensory block level was measured by assessing loss of pinprick discrimination until the level of sensory block was satisfactory for the operation.

### Measurements

A 20-gauge intraarterial line was inserted into the radial artery for continuous monitoring of the cardiac output, systolic blood pressure and heart rate. The FloTrac/Vigileo system was connected to the arterial line, and the transducer was adjusted to the level of the left atrium according to the instructions of the manufacturer[15]. The relevant variables were recorded before fluid preloading (baseline, T1), immediately after preloading completion (T2), after spinal anesthesia reached a peak sensory block of Th9 (T3), 15 min (T4), 30 min (T5), and 45 min (T6) post spinal block, and the end of operation (T7), respectively.

Hypotension was defined as a decrease in systolic blood pressure to <90 mm Hg or <75% of the baseline value, and was treated with an intravenous bolus of ephedrine (6 mg). Bolus was repeated if needed. Bradycardia was defined as heart rate <50 beats/min and was treated with intravenous atropine (0.5 mg).

### Statistical analysis

Data were expressed as mean±SD or number (percentage). The primary outcome variable was CO change. Sample size calculations indicated that inclusion of 15 patients per group would have 80% power at the 5% significance level to detect a difference in CO of 1.0 L/min between groups, assuming an SD of CO of 1.1 L/min (obtained from a pilot study). Secondary outcome variable was the incidence of hypotension. Continuous variables were compared with one-way ANOVA. Hemodynamic values over time were compared using analysis of variance for repeated measures. Post-hoc testing was performed with the Bonferroni method. Categorical variables were compared with the χ^2^ or Fisher exact test as appropriate. All analysis was performed using SPSS (Version 13.0) for Windows statistical software (SPSS Inc., Chicago, IL, USA). *P* < 0.05 was considered statistically significant.

## RESULTS

One patient in the control group was excluded from the study because of inadequate spinal anesthesia and required general anesthesia. Patient demographic characteristics were similar among the groups (*P* > 0.05). The main comorbid disease was hypertension in 9 patients in the control group, 6 in the RS group and 8 in the HES group (*P* > 0.05, ***Table 1***).

**Table 1:** Patient characteristics and baseline hemodynamic data

Parameters	Control (*n*=15)	HES (*n*=16)	RS (*n*=16)
Age(years)	72 ± 7	69 ± 8	71 ± 7
Weight(kg)	61 ± 7	62 ± 7	64 ± 8
Height(cm)	159 ± 9	161 ± 8	161 ± 7
Sex(M/F)	4/12	4/12	5/11
Hypertension, [*n* (%)]	9(60)	8(50)	6(38)
HR(beats/min)	79 ± 13	74 ± 9	76 ± 11
SBP(mmHg)	141.9 ± 15.4	144.9 ± 17.9	139.3 ± 17.0
CO(L/min)	5.3 ± 0.8	5.2 ± 0.6	4.9 ± 0.8

Values are mean±SD or number (percentage). CO: cardiac output; HES: the colloid group; HR: heart rate; RS: the crystalloid group; SBP: systolic blood pressure.

**Fig.1:**
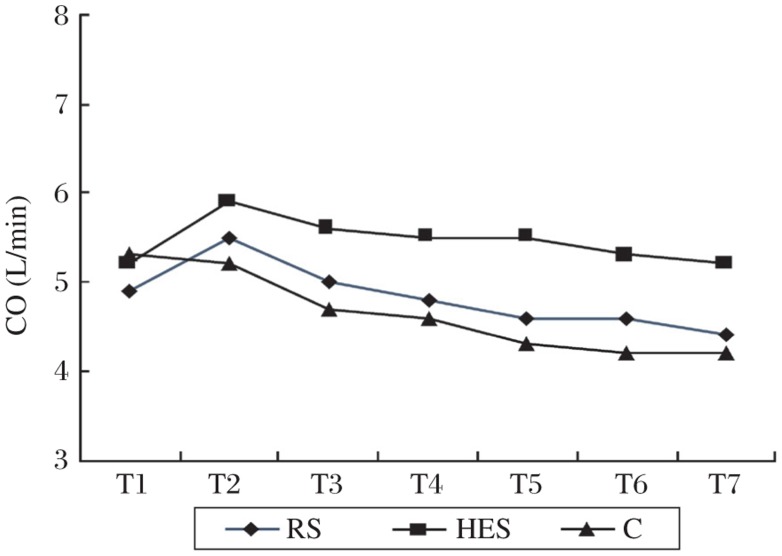
Cardiac output (CO) changes over time in control patients (C) and in patients who received an intravenous preload of 8 mL/kg of either lactated Ringer's solution (RS) or hydroxyethyl starch solution (HES).

**Fig. 2:**
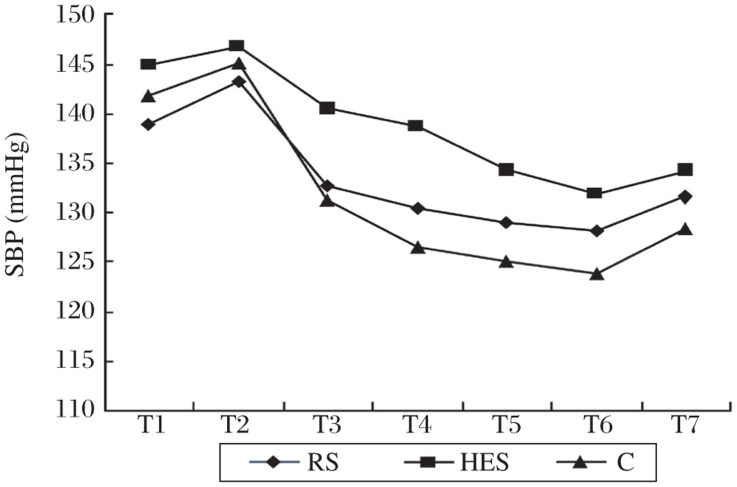
Systolic blood pressure (SBP) changes over time in control patients (C) and in patients who received an intravenous preload of 8 mL/kg of either lactated Ringer's solution (RS) or hydroxyethyl starch solution (HES).

**Fig. 3:**
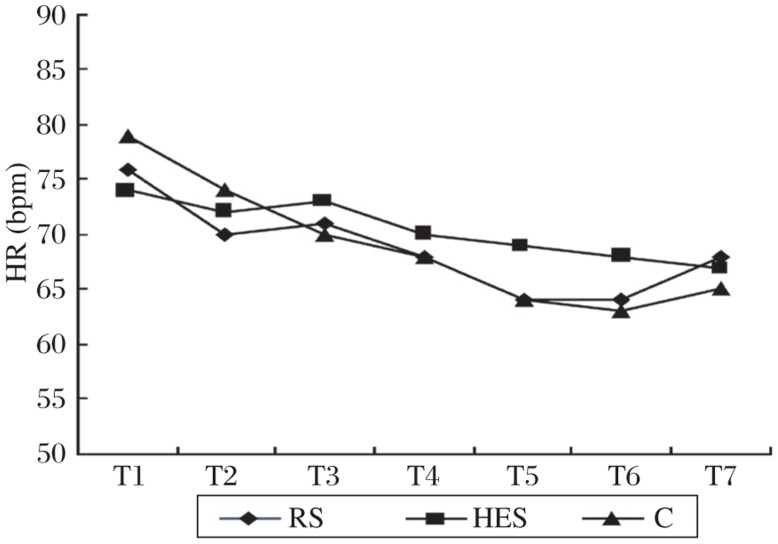
Heart rate changes over time in control patients (C) and in patients who received an intravenous preload of 8 mL/kg of either lactated Ringer's solution (RS) or hydroxyethyl starch solution (HES).

Changes in the cardiac output, systolic blood pressure and heart rate over time are shown in ***Table 2*** and ***Fig. 1***-***3***. Baseline hemodynamic variables were comparable among the groups (*P* > 0.05). Overall, there was a trend toward gradual decrease in cardiac output in the control group, while a trend toward an initial increase and a subsequent decrease in the cardiac output in both the RS and HES group. Repeated-measures ANOVA showed that the cardiac output at T2 was significantly higher than baseline value (T1) in both the RS and HES group (*P* < 0.01). The cardiac output remained significantly higher than baseline until 30 min after induction of spinal anesthesia in the HES group. In contrast, the CO was not different from baseline at any time during spinal anesthesia in the RS group. Intergroup comparison showed that the CO in the HES group was significantly higher than that in the RS and C groups at the corresponding time point during spinal anesthesia, but no significant difference existed between the RS and C groups (***Fig. 1***).

**Table 2:** Mean cardiac output in the three groups at different time-points

Group	Tl	T2	T3	T4	T5	T6	T7
Control	5.3 ± 0.9	5.2 ± 0.7	4.7 ± 0.8	4.6±0.8	4.3 ± 0.6	4.2 ± 0.7	4.2 ± 0.8
RS	4.9 ± 0.8	5.5 ± 0.6*	5.0±0.8	4.8±0.7	4.6 ± 0.6	4.6 ± 0.9	4.4±0.7
HES	5.2 ± 0.6	5.9 ± 0.7*	5.6±0.8*	5.5±0.8*	5.5 ± 0.7*	5.3 ± 0.7	5.2 ± 0.7

HES: the colloid group; RS: the crystalloid group; T1: before fluid administration; T2: after fluid administration; T3: after spinal anesthesia reached a peak sensory block of Th9; T4: 15 min after spinal block; T5: 30 min after spinal block; T6: 45 min after spinal block; T7: the end of surgery. *P < 0.01 compared with T1.

(mean±SD)

A change trend similar to that of the cardiac output was found with systolic blood pressure. However, there were no significant differences from baseline during spinal anesthesia within each group, or at the corresponding time points among three groups (***Fig. 2***). Hypotension occurred in 3 patients in the C group and one each in the RS group and HES group, respectively (*P* = 0.362). In addition, there was a nonsignificant trend toward decrease in the heart rate after spinal anesthesia in all patients, with no significant difference among the three groups (***Fig. 3***).

## DISCUSSION

The present study showed that fluid preload alleviated the trend toward decrease in the cardiac output and systolic blood pressure after induction of spinal anesthesia in elderly patients. Furthermore, this effect was more evident in patients who received colloid as preload than those who received crystalloid solution.

Total hip replacement is usually performed in elderly patients who often have functional limitations and concomitant diseases, which compromise cardiovascular stability, leading to decreased organ perfusion and subsequently to organ dysfunction or cardiovascular complications[16]. Although routine blood pressure and heart rate measurements are of value in assessing the safety of anesthetic technique, cardiac output changes may be more important in terms of the goal of maintaining organ perfusion because the change in cardiac output has been shown to correlate better with tissue blood flow than blood pressure[17]–[20]. In addition, an increased systemic vascular resistance (SVR) in elderly patients could also render blood pressure a poor indicator of cardiac output and organ flow[21]. We therefore decided to investigate the effects of fluid preload on cardiac output changes associated with spinal anesthesia in elderly patients in this study.

Measurements of CO have previously been assessed by thermodilution, dye dilution, or Doppler ultrasound. These methods, however, may bring certain disadvantages with regard to their use during anesthesia. For example, thermodilution or dye dilution techniques require catheterization of the pulmonary artery, which may incur serious complications and limit their routine use[22]. Transthoracic Doppler echocardiography is operator-dependent and time-consuming and cannot be used for continuous measurements[23]. On the other hand, the FloTrac/Vigileo system, which is based on the standard deviation of the pulse pressure waveform, is minimally invasive, can be easily applied and may provide continuous real-time cardiac output data. Studies have confirmed a good agreement between continuous cardiac output measurements using FloTrac/Vigileo system with other methods including the “gold standard” pulmonary artery thermodilution in numerous clinical situations[24]–[27]. Therefore, the use of FloTrac/Vigileo system offers the occasion to conveniently detect immediate cardiac output changes and then improves hemodynamic management in elderly patients during anesthesia.

Hypotension results in inadequate organ perfusion and can ultimately end with the loss of consciousness and cardiovascular collapse, particularly in elderly patients. Induction of spinal anesthesia induces sympathetic block, which causes blood pressure to fall as a result of decreased systemic vascular resistance and cardiac output, the latter being secondary to reduced venous return and sometimes decreased heart rate[21]. Our results also reflected this tendency (***Fig. 2***). Therefore, optimizing cardiac preload may be fundamental to preventing spinal hypotension and organ dysfunction in elderly patients. However, to date, there are many literatures debating the type and amount of fluid preload[11]–[13]. The present study showed that a fluid preload of 8 mL/kg increased cardiac output. The increased cardiac output should be attributed to increase in stroke volume (i.e., cardiac preload) because heart rate did not increase corresponding at the same time. Furthermore, in accordance with a previous study[28], colloid preload was more effective than crystalloids for maintaining cardiac output. This is not surprising because colloid increases more intravascular volume and stays in the vascular space longer due to the larger molecular weight compared with crystalloid. As a result, blood pressure tended to decrease less in patients receiving colloid preload, although it did not reach statistical significance.

Of note, the incidence of hypotension was similar among the three groups in this study. Other studies have shown that fluid preload reduces the incidence of spinal-related hypotension[29]. However, the lack of significance in the incidence of hypotension may be the result of a type II statistical error due to the small sample in this study. Additionally, an alternative explanation may be the relatively lower volume of fluid preload in our elderly patients as compared with younger patients in other studies (8 mL/kg *vs* 15-20 mL/kg)[12],[30]. The volume of 8 mL/kg for fluid preload chosen in this study was mainly based on the clinical concern that more fluid preloading may put the elderly at increased risk of cardiovascular complications and pulmonary oedema[9]. A further larger prospective study then is needed to better clarify the efficacy of fluid preloading and to evaluate the optimal dose for the prevention of spinal hypotension. However, despite colloid preload at the volume studied did not significantly prevent spinal hypotension, the increase in CO should be beneficial for elderly patients during anesthesia as discussed above.

In conclusion, our data suggest that use of moderate volume of colloid preload is more effective than crystalloids in maintaining the CO and hemodynamic stability in elderly patients.
